# Fecal microbiome composition in neonates with or without urinary tract infection

**DOI:** 10.1007/s00467-024-06612-1

**Published:** 2024-11-28

**Authors:** Hilla Bahat, Michal Paret, Atara Uzan, Hodaya Klainer, Efrat Sharon, Sondra Turjeman, Omry Koren, Michael Goldman, Ilan Youngster

**Affiliations:** 1 Department of Pediatrics, Shamir Medical Center, 70300 Zerifin, Israel; 2 Faculty of Medical & Health Sciences, Tel Aviv University, Tel Aviv, Israel; 3 The Center for Microbiome Research, Shamir Medical Center, Zerifin, Israel; 4 Azrieli Faculty of Medicine, Bar-Ilan University, Safed, Israel

**Keywords:** Fecal microbiota, Infant, Urinary tract infection

## Abstract

**Background:**

Most infants with febrile urinary tract infection (UTI) do not have an underlying anatomical risk factor. Thus, other non-anatomical risk factors should be considered. Since the most common pathogens arise from the fecal microbiota, our aim was to investigate whether the gut microbiota composition differs between febrile infants younger than 2 months with or without UTI.

**Methods:**

In this prospective, case–control, pilot study, we performed 16S ribosomal ribonucleic acid amplicon sequencing to characterize gut microbiota of febrile neonates with and without UTI admitted to the pediatric ward at Shamir Medical Center between February 2019 and May 2021.

**Results:**

The study cohort included 42 febrile neonates: 17 with and 25 without febrile UTI. We found a significant difference in beta diversity (i.e. between-sample/study group similarity indices) between the UTI and non-UTI group (*p* = 0.016). There were also distinct differences in the relative abundance of the 20 most prevalent genera. Furthermore, several genera were significantly enriched in the UTI group, with others dominating the non-UTI group. Streptococci were underrepresented in the UTI group. There was no difference in alpha diversity (i.e. within-sample diversity/richness) between groups.

**Conclusion:**

Febrile neonates with UTI have a different fecal microbiota composition (beta-diversity), but not alpha diversity, in comparison to febrile neonates without UTI. A larger study is warranted to confirm these findings and their potential applications.

**Graphical abstract:**

**Figure:**
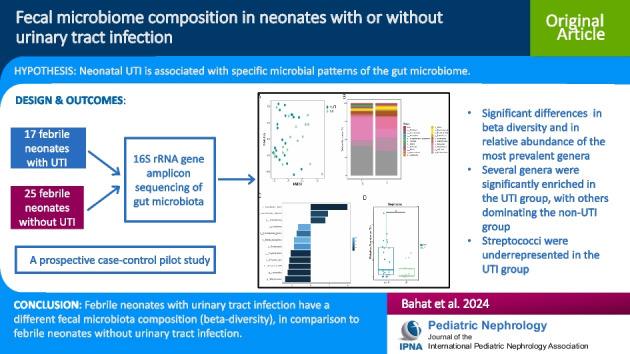
A higher resolution version of the Graphical abstract is available as Supplementary information

**Supplementary Information:**

The online version contains supplementary material available at 10.1007/s00467-024-06612-1.

## Introduction

Urinary tract infection (UTI) is the most common serious bacterial infection in neonates and young infants [1]. Managing UTIs in the neonatal period involves parenteral empiric antibiotic therapy and subsequently implementing microorganism-specific therapy. Treatment should be initiated promptly ensuring broad coverage for the most probable pathogens [2]. In most infants with UTI, no risk factor is identified, and high grade vesicoureteral reflux (VUR) is present in only about 30% of cases [1].

The human gut microbiome refers to the diverse community of microorganisms (bacteria, viruses and fungi) that inhabit the gastrointestinal tract. The composition of the gut microbiome is influenced by factors such as genetics, diet, lifestyle, environmental and medication exposures, while disruption in its equilibrium, known as dysbiosis, has been associated with various health conditions [3, 4]. The microbiome evolves throughout life, maintaining a balance with the host’s immune system, affected and shaped by both internal factors and external influences post-birth. The microbial composition plays a direct role in protection against excessive growth of pathogens and regulates the balance between inflammation and systemic immune function [5].

The neonatal gut microbiome, established at birth and rapidly evolving in early infancy, is influenced by various factors including, but not limited to, mode of birth, nutrition, prematurity, antibiotic exposure, and maternal microbiota [6]. In the presence of infection, dysbiosis can occur in the infant's microbiome, marked by the proliferation of pathogenic species that disrupt the delicate balance between the host and microbiome. For instance, there is growing recognition that necrotizing enterocolitis is linked to disruptions in the intestinal immune balance and disruptions of the normal colonization pattern of the gastrointestinal system [7].

Recently, there has been an increased interest in the relation between the intestinal microbiome and several disorders, including kidney disease [8]. Since the most common pathogens causing UTI arise from the fecal microbiota, and similar strains have been isolated from the urine and feces of patients with UTI [9], specific fecal microbiome characteristics can potentially be related to an increased risk of UTI [10–12]. To the best of our knowledge, gut microbiome characteristics in neonates with febrile UTI have not been studied before.

In the present study we examined the differences in fecal microbiome composition between febrile neonates with and without a UTI.

## Patients and methods

In this prospective, case–control, pilot study, we recruited febrile neonates admitted to the pediatric ward at Shamir Medical Center, Israel between February 2019 and May 2021, after obtaining parental written informed consent. The inclusion criteria were: age 1 week to 2 months on admission, having a urine culture obtained by a urinary catheter or suprapubic aspiration, and having a fecal sample collected within 48 h of antibiotic treatment initiation. Patients were excluded if they had a history of a previous UTI, received prophylactic antibiotic treatment, or were treated with immunosuppressive medications.

At enrollment, a questionnaire was completed including maternal and infant demographics and medical history. Data regarding laboratory workup, diagnostic imaging, and final diagnosis was collected after discharge. UTI was diagnosed in the presence of a positive urine culture with growth of ≥ 50,000 colony-forming units/ml of a single known urinary pathogen.

The study was approved by the local ethics board at Shamir Medical center and adheres to the Declaration of Helsinki, approval number 0201–18-ASF.

### Sample collection and analysis

Fresh stool samples were collected from diapers after study participation consent, within 48 h of antibiotic initiation. Samples were frozen within 4 h to preserve microbial diversity and stored at –80 °C until further analyses. All samples were processed in a single batch at the end of recruitment. DNA was extracted from infant's stool samples using the Invitrogen Microbiome DNA Purification kit (Thermo Fisher) according to the manufacturer's instructions, following a bead beating (BioSpec) step for 2 min. Following DNA extraction, the V4 variable region of the bacterial 16S rRNA amplicon was amplified by polymerase chain reaction (PCR) using barcoded 515F primers and non-barcoded 806R primers [13]. PCR reactions were carried out with the PrimeSTAR Max Taq polymerase enzyme (Takara) for 30 cycles of denaturation (95 °C, 10 s), annealing (55 °C, 5 s) and extension (72 °C, 20 s), and a final elongation at 72 °C (1 min). Products were purified using AMPure magnetic beads (Beckman Coulter) and quantified using the Pico-green dsDNA quantitation kit (Invitrogen). Samples were pooled at equal concentrations (50 ng/µl), loaded on 2% E-Gel (Thermo Fisher) and purified using the NucleoSpin Gel and PCR Clean-up (Macherey–Nagel). Purified products were sequenced using the Illumina MiSeq platform (Genomic Center, Azrieli Faculty of Medicine, BIU, Israel). FASTQ files were processed using FastQC (version 0.11.5) to perform quality control on the raw sequences. The QIIME2 pipeline [14] (version qiime2-2020.8) was used on 16S rRNA amplicon raw sequences from microbial communities. The pipeline includes: importing the files, demultiplexing using the q2-demux plugin, denoising, dereplication and removing chimeras using the DADA2 [15] algorithm, *de novo* amplicon sequence variant (ASV) clustering using vsearch with 97% identity, and assigning taxonomy using the classify-sklearn naïve base classifier against GreenGenes [16] database. ASVs were named based upon the finest resolution allowed by the classifier, but species-level assignments of 16S rRNA amplicon sequence data may be biased. After the QIIME2 pipeline, downstream analysis was performed using the phyloseq [17] (version 1.40.0) R/bioconductor package for handling and analysis of high-throughput phylogenetic sequence data. The taxonomy was first cleaned and filtered, removing empty taxa. The samples were then rarefied (using the rarefy_even_depth function) to a minimum sequence depth of 1,500 and scaled to relative abundance. Further analysis included 42 samples, 17 samples of UTI patients and 25 samples of febrile non-UTI patients. ASVs were filtered out if they appeared in less than 4 samples.

### Statistical analysis

We aimed to recruit 40 participants based on estimates derived from an expected 20% difference in alpha diversity indices in fecal microbiome between the groups and a 90% power.

The clinical and demographic characteristics were analyzed using Mann–Whitney U tests, and Chi-square/Fisher’s exact test. In order to look for associations between the metadata obtained and the abundances of the ASVs summarized at different taxonomic levels, we used Spearman rank correlations for the continuous metadata and ANOVA for the categorical metadata. We corrected for multiple comparisons using false discovery rate (FDR) corrections.

### Profile comparisons

To answer the study question, we calculated and compared alpha (estimate_richness function, Faith’s PD- in the twbattaglia/btools package) [18] and beta diversity (distance function; unweighted and weighted UniFrac) [19] across the different groups of samples. Plots were generated using ggplot2 [https://cran.r-project.org/web/packages/ggplot2/citation.html (version 3.4.0)] and pheatmap [https://cran.r-project.org/web/packages/pheatmap/index.html (version 1.0.12)].

To assess microbial differential taxon abundance, the DESeq2 (version 1.36.0) [20], R/bioconductor package was used. Rarified scaled ASVs were labeled by the lowest assigned taxa level possible and were summarized per taxa. The groups were compared, and significant taxa identified (*p* adjusted values < 0.05 and |log2foldchange|≥ 0.58).

## Results

**Fig. 1:**
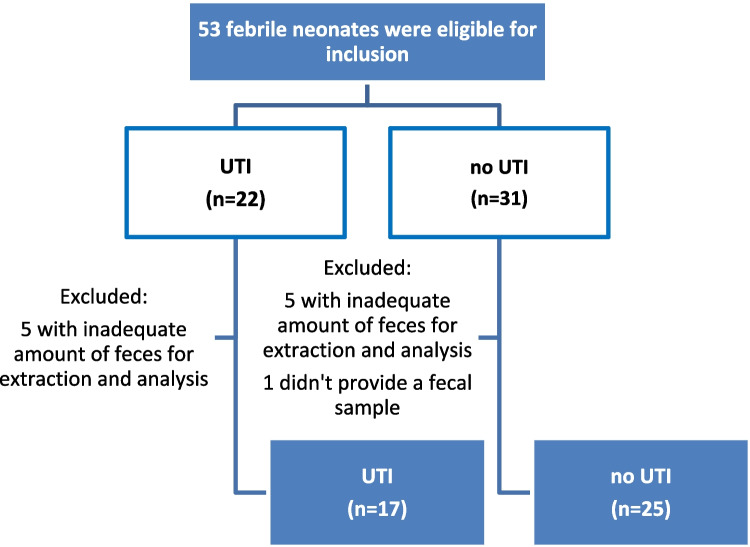
Flow chart of patient selection

**Fig. 2:**
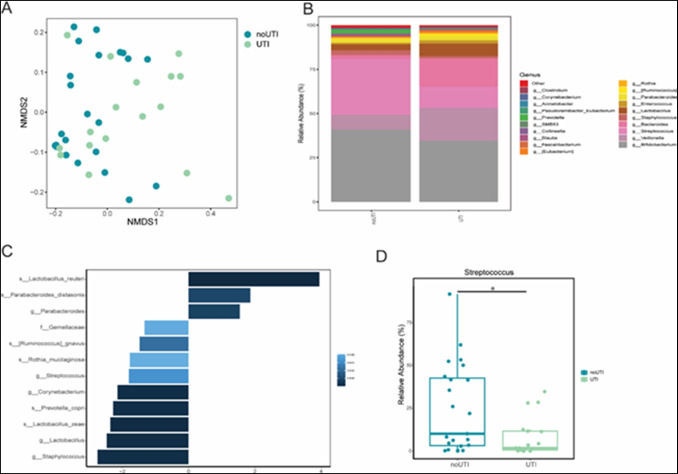
Alterations in gut bacterial composition in infants following urinary tract infection. 16S rRNA sequencing was performed to evaluate changes in gut bacterial composition. **A** Non-metric multidimensional scaling (NMDS) based on Weighted UniFrac distances between UTI (green) and nonUTI (blue) infants showing significant difference in bacterial clustering (*p*-value = 0.016). **B** Relative taxonomic composition based on 20 most abundant genera of UTI and nonUTI groups. **C** DESeq paired analysis identifying differentially abundant bacteria between UTI and non UTI infants. Log2fold changes greater than zero indicated an increase in the relevant bacteria in the UTI group, whereas log2fold changes less than zero indicated an increase in the relevant bacteria among the nonUTI group. **D** Box plot representing significantly higher levels of *Streptococcus* among the nonUTI group compared to the UTI group. (*P < 0.05, **P < 0.01, ***P < 0.001 and ****P < 0.0001; minimum **15** samples in each group)

Fifty-three infants were eligible for inclusion. Eleven were excluded due to an inadequate amount of feces for extraction and analysis. The final study cohort (following sequencing quality control) included 42 febrile neonates: 17 cases with UTI and 25 controls without UTI (Fig. 1). The characteristics of the study cohort are shown in Table 1. The differences between the groups were the expected increase in inflammatory markers (leukocytosis and elevated C-reactive protein) in the UTI group (*P* < 0.001), and the different antibiotic treatment prescribed after admission. In the control group, only one patient had a presumed bacterial infection (acute otitis media), 12 had positive viral panels (9 with enterovirus, 1 with influenza, 1 with parainfluenza and 1 with adenovirus), and the rest had fever with negative blood, cerebrospinal fluid, stool, and urine cultures. In the UTI group, one of the 15 infants that performed a voiding cystourethrography (VCUG) had significant (grade 4) VUR. Sixteen infants had *E. coli* UTI and pyuria, and one had *Enterococcus faecalis* UTI with a negative dipstick, elevated inflammatory markers, and high grade VUR (and thus was considered as true UTI in the absence of pyuria). Weighted UniFrac analysis, assessed using non-metric multidimensional scaling, showed a significant difference in clustering (*p* = 0.016) between the UTI and non-UTI groups (Fig. 2A). The relative abundance of the 20 most abundant genera between the UTI and non-UTI groups showed differences in gut bacterial composition (Fig. 2B). Furthermore, taxonomic analysis, based on the relative abundance at the species level, revealed several bacteria that were significantly enriched in the UTI group: *Lactobacillus reuteri, Bacteroides, Parabacteroides distasonis, Parabacteroides merdae,* and* Bacteroides uniformis*. Others were prominently represented in the non-UTI group (Fig. 2C). Most notably, *Streptococcus* was almost exclusively detected in the control group (Fig. 2D). No significant differences in alpha diversity were detected between the groups (Supplementary Fig. 1), nor were any differences in alpha or beta diversity detected between study groups based on metadata-specific analysis including delivery mode, sex, age and nutrition (data not shown).

**Table 1:** Cohort characteristics

	Urinary tract infection *n* = 17	Controls *n* = 25	*P* value
Age (weeks)^a^	3 (2–5.5)	4 (2.5–5.25)	0.6
Gestational age (weeks)^a^	39 (38.5–40)	39 (38–41)	0.77
Birth weight (kg)^a^	3.42 (3.28–3.57)	3.17 (2.8–3.6)	0.12
White Blood Cells (k/µl)^a^	18.8 (16.9–21.75)	11.3 (6.9–13.6)	< 0.001
C-Reactive Protein (mg/L)^a^	51.9 (17.5–106.5)	9.9 (3.4–27.2)	< 0.001
Sex:			1
Male	9	14	
Female	8	11	
Nutrition:			0.73
Breast milk	4	8	
Formula/combined	13	17	
Deivery mode:			1
Vaginal delivery	15	21	
Cesarean section	2	4	
Antibiotic treatment^b^:			< 0.001
Ampicillin and gentamicin	15	1	
Ampicillin and cefotaxime	2	23	

^a^ Presented as median (IQR)

^b^ one neonate in the control group was treated with ceftriaxone

## Discussion

Most infants with febrile UTI do not have an underlying anatomical risk factor like VUR [1]; thus, other non-anatomical risk factors should be considered. Since the most common pathogens causing UTI arise from the fecal microbiota [9], we speculated that there might be a specific microbial pattern of the gut microbiome that will be associated with UTI. Our study shows that neonates with febrile UTI have a distinct gut microbiome composition, compared to febrile neonates without bacterial infection. *Lactobacillus reuteri, Bacteroides, Parabacteroides distasonis, Parabacteroides merdae* and* Bacteroides uniformis* were all overrepresented, while *Streptococcus* was underrepresented in the UTI group. To the best of our knowledge, this is the first study assessing fecal microbiome composition in neonates and young infants with UTI. Since probiotics and fecal microbiota transplantation (FMT) has been shown to prevent and treat recurrent infections [21–23], potential clinical implications of our study include microbiome targeted interventions, once gut microbiota imbalance and lack of specific strains in the gut of infants with UTI will be confirmed in larger future studies.

Even in other age groups, the literature about the association between fecal microbiome and UTI is sparse, especially among children. Paalanne et al. compared fecal microbiomes of 37 older children with febrile UTI (mean age 20 months) with healthy controls and found that the genus *Enterobacter* was more prominent in the UTI group, as opposed to *Peptostreptococcaceae* in the controls. There were no differences at the phylum level [10]. Magruder et al. studied the fecal microbiome of kidney transplant recipients with and without UTI, and found that gut abundance of uropathogens was associated with UTI [11], and that the relative abundance of *Faecalibacterium* and *Romboutsia* were associated with a decreased risk of *Enterobacteriaceae* UTI [12]. Urakami et al. showed that the gut microbiota of older infants with a first febrile UTI was characterized by a lower alpha diversity, a lower proportion of *B. fragilis* and a higher proportion of *Escherichia-Shigella* when compared to healthy controls [24].

Antibiotic treatment was found to transiently change the intestinal microbiome in infants [25, 26]. Baldi et al. [25] showed a transient reduction in alpha diversity of the intestinal microbiome in infants who received antibiotics within the previous week, and enrichment for *Enterococcus* and *Escherichia/ Shigella*. This effect was not seen seen in children who received antibiotics more than a week before fecal sampling. Similarily, Yassour et al. [26]. found a decreased microbial diversity and increased short-term composition changes in the gut microbiomes of antibiotic-treated children, that returned to baseline within one month. In our study we only assessed the infant gut microbome of prior to treatment, but it would be interesting to study longitudinally if this effect of "return to baseline" after treatment is similar among infants with or without UTI.

Our study has limitations. First, the sample size is limited, and larger studies are warranted. Second, the study groups were treated differently and the fecal samples were collected up to 48 h after starting antibiotic treatment, which can potentially influence the fecal microbiome. However, since bacterial viability is not a prerequisite for 16S sequencing, it is not expected to influence results early in the treatment course. Furthermore, both study groups were antibiotically treated as part of neonatal fever workup, and thus this should not influence comparative analyses. Another limitation might be the lack of healthy subjects as a comparison group. However, we chose febrile neonates without UTI as the control group to highlight the specific impact of UTI, and not the influence of an acute infection, that has been shown previously to change the fecal microbiome in neonates [27]. Lastly, due to the limited sample size we only had one neonate with a UTI caused by pathogens other than *E. coli*, precluding subanalyses. Future work should investigate whether the gut microbiome is associated with different urinary pathogens.

In summary, we found that neonates with UTI have some specific fecal microbiome characteristics. A larger study is warranted to confirm our findings. Potential future implication of these findings, if validated, include new insights into factors contributing to development of febrile UTI in neonates, new modalities for early culture-independent diagnostics, and development of preventive measures.

## Supplementary Information

Below is the link to the electronic supplementary material.

Graphical abstract (PPTX 153 KB)

Supplementary file2 (DOCX 140 KB)

## Data Availability

The study data will be made available by request from the corresponding author.

## References

[1] Hoberman A, Keren R (2009) Antimicrobial prophylaxis for urinary tract infection in children. N Engl J Med 361:1804–1806. 10.1056/nejme0907623 19864680 10.1056/NEJMe0907623

[2] Pantell RH, Roberts KB, Adams WG, Dreyer BP, Kuppermann N, O’Leary ST et al (2021) Clinical practice guideline: evaluation and management of well-appearing febrile infants 8 to 60 days old. Pediatrics 148:602–603. 10.1001/jamapediatrics.2022.0066 10.1542/peds.2021-052228 34281996

[3] Wilson AS, Koller KR, Ramaboli MC et al (2020) Diet and the human gut microbiome: an international review. Dig Dis Sci 65:723–740. 10.1007/s10620-020-06112-w 32060812 10.1007/s10620-020-06112-w PMC7117800

[4] Cani PD (2018) Human gut microbiome: hopes, threats and promises. Gut 67:1716–1725. 10.1136/gutjnl-2018-316723 29934437 10.1136/gutjnl-2018-316723 PMC6109275

[5] Lambring CB, Siraj S, Patel K et al (2019) Impact of the microbiome on the immune system. Crit Rev Immunol 39:313–328. 10.1615/CritRevImmunol.2019033233 32422014 10.1615/CritRevImmunol.2019033233 PMC7362776

[6] Gritz EC, Bhandari V (2015) The human neonatal gut microbiome: a brief review. Front Pediatr 3:17. 10.3389/fped.2015.00017 25798435 10.3389/fped.2015.00017 PMC4350424

[7] Carlisle EM, Morowitz MJ (2013) The intestinal microbiome and necrotizing enterocolitis. Curr Opin Pediatr 25:382–387. 10.1097/MOP.0B013E3283600E91 23657248 10.1097/MOP.0b013e3283600e91

[8] Ahlawat S, Asha SKK (2021) Gut–organ axis: a microbial outreach and networking. Lett Appl Microbiol 72:636–668. 10.1111/lam.13333 32472555 10.1111/lam.13333

[9] Yamamoto S, Tsukamoto T, Terai A, Kurazono H, Takeda Y, Yoshida O (1997) Genetic evidence supporting the fecal-perineal-urethral hypothesis in cystitis caused by Escherichia coli. J Urol 157:1127–1129 9072556

[10] Paalanne N, Husso A, Salo J et al (2018) Intestinal microbiome as a risk factor for urinary tract infections in children. Eur J Clin Microbiol Infect Dis 37:1881–1891. 10.1007/s10096-018-3322-7 30006660 10.1007/s10096-018-3322-7

[11] Magruder M, Sholi AN, Gong C et al (2019) Gut uropathogen abundance is a risk factor for development of bacteriuria and urinary tract infection. Nat Commun 10:5521. 10.1038/s41467-019-13467-w 31797927 10.1038/s41467-019-13467-w PMC6893017

[12] Magruder M, Edusei E, Zhang L et al (2020) Gut commensal microbiota and decreased risk for Enterobacteriaceae bacteriuria and urinary tract infection. Gut Microbes 12:1805281. 10.1080/19490976.2020.1805281 32865119 10.1080/19490976.2020.1805281 PMC7524266

[13] Caporaso JG, Lauber CL, Walters WA et al (2012) Ultra-high-throughput microbial community analysis on the Illumina HiSeq and MiSeq platforms. ISME J 6:1621–1624. 10.1038/ismej.2012.8 22402401 10.1038/ismej.2012.8 PMC3400413

[14] Bolyen E, Rideout JR, Dillon MR et al (2019) Reproducible, interactive, scalable and extensible microbiome data science using QIIME 2. Nat Biotechnol 37:852–857 31341288 10.1038/s41587-019-0209-9 PMC7015180

[15] Callahan BJ, McMurdie PJ, Rosen MJ, Han AW, Johnson AJ, Holmes SP (2016) DADA2: High resolution sample inference from Illumina amplicon data. Nat Methods 13:581–583. 10.1038/nmeth.3869 27214047 10.1038/nmeth.3869 PMC4927377

[16] DeSantis TZ, Hugenholtz P, Larsen N et al (2006) Greengenes, a chimera-checked 16S rRNA gene database and workbench compatible with ARB. Appl Environ Microbiol 72:5069–5072. 10.1128/AEM.03006-05 16820507 10.1128/AEM.03006-05 PMC1489311

[17] McMurdie PJ, Holmes S (2013) Phyloseq: an R package for reproducible interactive analysis and graphics of microbiome census data. PLoS One 8:e61217. 10.1371/journal.pone.0061217 23630581 10.1371/journal.pone.0061217 PMC3632530

[18] Faith DP (1992) Conservation evaluation and phylogenetic diversity. Biol Conserv 61:1–10

[19] Lozupone C, Knight R (2005) UniFrac: a new phylogenetic method for comparing microbial communities. Appl Environ Microbiol 71:8228–8235. 10.1128/AEM.71.12.8228-8235.2005 16332807 10.1128/AEM.71.12.8228-8235.2005 PMC1317376

[20] Love MI, Huber W, Anders S (2014) Moderated estimation of fold change and dispersion for RNA-seq data with DESeq2. Genome Biol 15:550. 10.1186/s13059-014-0550-8 25516281 10.1186/s13059-014-0550-8 PMC4302049

[21] Tan GSE, Tay HL, Tan SH et al (2020) Gut microbiota modulation: implications for infection control and antimicrobial stewardship. Adv Ther 37:4054–4067. 10.1007/s12325-020-01458-z 32767183 10.1007/s12325-020-01458-z PMC7412295

[22] Li KL, Wang BZ, Li ZP et al (2019) Alterations of intestinal flora and the effects of probiotics in children with recurrent respiratory tract infection. World J Pediatr 15:255–261. 10.1007/s12519-019-00248-0 31020541 10.1007/s12519-019-00248-0 PMC6597592

[23] Ugwu OPC, Alum EU, Ben OM, Obeagu EI (2024) Mechanisms of microbiota modulation: Implications for health, disease, and therapeutic interventions. Med (United States) 103:E38088. 10.1097/MD.0000000000038088 10.1097/MD.0000000000038088 PMC11081615 38728472

[24] Urakami C, Yamanouchi S, Kimata T et al (2023) Abnormal development of microbiota may be a risk factor for febrile urinary tract infection in infancy. Microorganisms 11:2574. 10.3390/microorganisms11102574 37894232 10.3390/microorganisms11102574 PMC10609410

[25] Baldi A, Braat S, Imrul Hasan M et al (2024) Community use of oral antibiotics transiently reprofiles the intestinal microbiome in young Bangladeshi children. Nat Commun 15:6980. 10.1038/s41467-024-51326-5 39143045 10.1038/s41467-024-51326-5 PMC11324872

[26] Yassour M, Vatanen T, Siljander H, Hämäläinen AM, Härkönen T, Ryhänen SJ, Franzosa EA, Vlamakis H, Huttenhower C, Gevers D, Lander E, Knip D, DIABIMMUNE Study Group, Xavier RJ (2016) Natural history of the infant gut microbiome and impact of antibiotic treatments on strain-level diversity and stability. Sci Transl Med 8:343ra81. 10.1126/scitranslmed.aad0917 27306663 10.1126/scitranslmed.aad0917 PMC5032909

[27] Berrington JE, Stewart CJ, Cummings SP, Embleton ND (2014) The neonatal bowel microbiome in health and infection. Curr Opin Infect Dis 27:236–243. 10.1097/QCO.0000000000000061 24751892 10.1097/QCO.0000000000000061

