# Supplementary material for: Fecal microbiome composition in neonates with or without urinary tract infection
Source: Pediatr Nephrol. 2024 Nov 28;40(4):1015–21. doi: 10.1007/s00467-024-06612-1 (PMC11885367; doi:10.1007/s00467-024-06612-1)
Supplement: Supplementary file 1 — Graphical abstract (PPTX 153 KB) [file 467_2024_6612_MOESM1_ESM.pptx]

## Slide 1
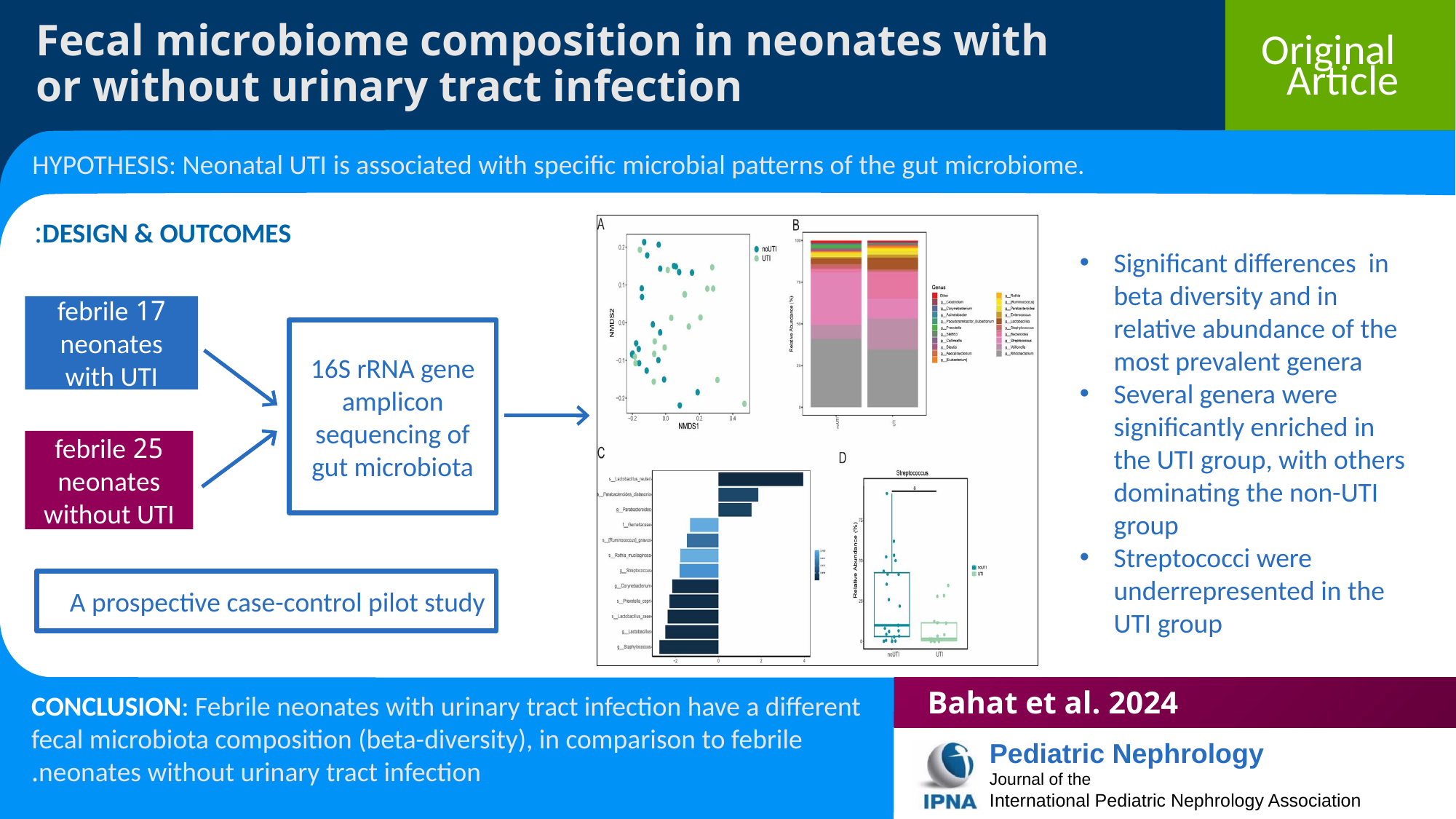

Fecal microbiome composition in neonates with or without urinary tract infection
HYPOTHESIS: Neonatal UTI is associated with specific microbial patterns of the gut microbiome.
DESIGN & OUTCOMES:
Significant differences in beta diversity and in relative abundance of the most prevalent genera
Several genera were significantly enriched in the UTI group, with others dominating the non-UTI group
Streptococci were underrepresented in the UTI group
17 febrile neonates with UTI
16S rRNA gene amplicon sequencing of gut microbiota
25 febrile neonates without UTI
A prospective case-control pilot study
Bahat et al. 2024
CONCLUSION: Febrile neonates with urinary tract infection have a different fecal microbiota composition (beta-diversity), in comparison to febrile neonates without urinary tract infection.
