# Supplementary figures and images for: Fecal microbiome composition in neonates with or without urinary tract infection
Source: Pediatr Nephrol. 2024 Nov 28;40(4):1015–21. doi: 10.1007/s00467-024-06612-1 (PMC11885367; doi:10.1007/s00467-024-06612-1)

Supplementary Figure 1: Alpha diversity of UTI and non-UTI groups


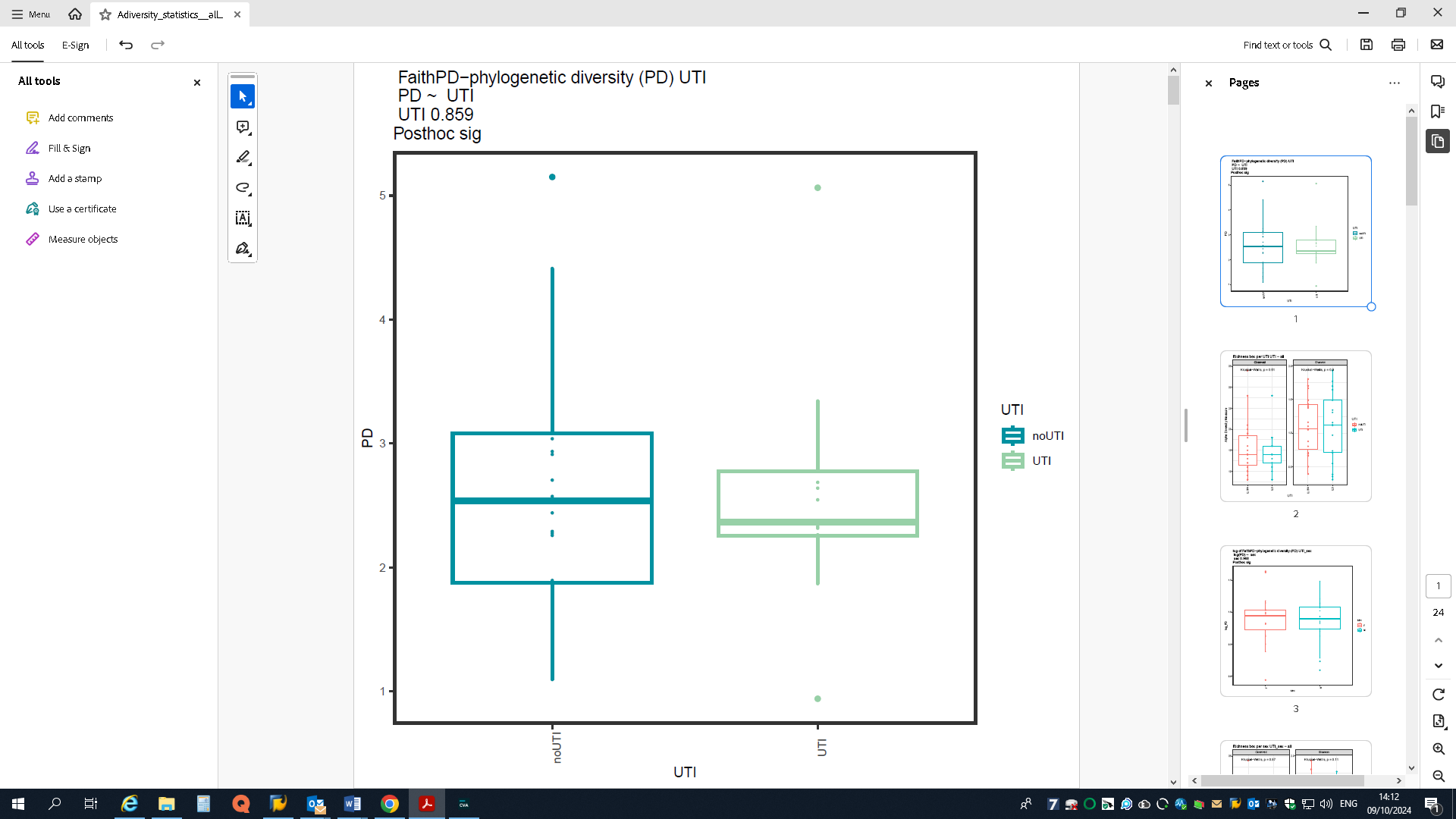

Supplement: Supplementary file 2 — Supplementary file2 (DOCX 140 KB) [file 467_2024_6612_MOESM2_ESM.docx]
